# CREAMMIST: an integrative probabilistic database for cancer drug response prediction

**DOI:** 10.1093/nar/gkac911

**Published:** 2022-10-19

**Authors:** Hatairat Yingtaweesittikul, Jiaxi Wu, Aanchal Mongia, Rafael Peres, Karrie Ko, Niranjan Nagarajan, Chayaporn Suphavilai

**Affiliations:** Advanced Research Center for Computational Simulation, Faculty of Science, Chiang Mai University, Chiang Mai, Thailand; Genome Institute of Singapore, A*STAR, Singapore, Singapore; Genome Institute of Singapore, A*STAR, Singapore, Singapore; Genome Institute of Singapore, A*STAR, Singapore, Singapore; Genome Institute of Singapore, A*STAR, Singapore, Singapore; Genome Institute of Singapore, A*STAR, Singapore, Singapore; Genome Institute of Singapore, A*STAR, Singapore, Singapore

## Abstract

Extensive *in vitro* cancer drug screening datasets have enabled scientists to identify biomarkers and develop machine learning models for predicting drug sensitivity. While most advancements have focused on omics profiles, cancer drug sensitivity scores precalculated by the original sources are often used as-is, without consideration for variabilities between studies. It is well-known that significant inconsistencies exist between the drug sensitivity scores across datasets due to differences in experimental setups and preprocessing methods used to obtain the sensitivity scores. As a result, many studies opt to focus only on a single dataset, leading to underutilization of available data and a limited interpretation of cancer pharmacogenomics analysis. To overcome these caveats, we have developed CREAMMIST (https://creammist.mtms.dev), an integrative database that enables users to obtain an integrative dose-response curve, to capture uncertainty (or high certainty when multiple datasets well align) across five widely used cancer cell-line drug–response datasets. We utilized the Bayesian framework to systematically integrate all available dose-response values across datasets (>14 millions dose-response data points). CREAMMIST provides easy-to-use statistics derived from the integrative dose-response curves for various downstream analyses such as identifying biomarkers, selecting drug concentrations for experiments, and training robust machine learning models.

## Graphical Abstract

**Graphical Abstract:**
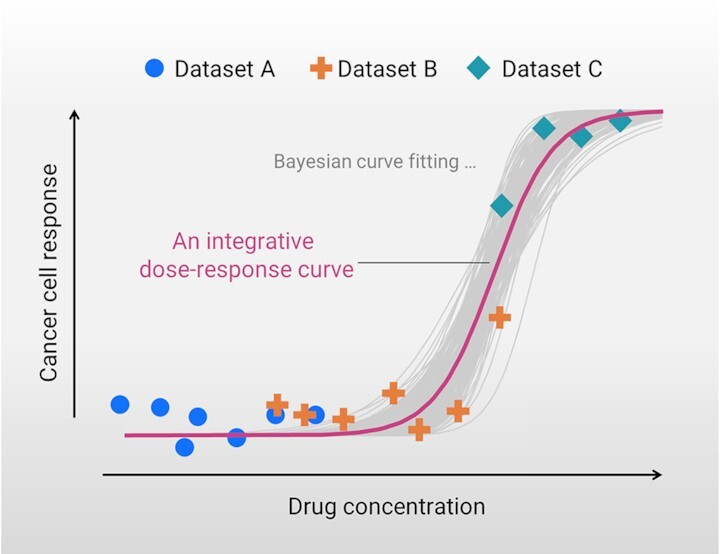
CREAMMIST is an integrative probabilistic database for cancer drug response prediction and pharmacogenomics analysis. It combines widely used in vitro cancer drug screening datasets and provides integrative dose-response curves.

## INTRODUCTION

Cancer is a complex disease in which inter- and intra-tumor heterogeneity can impact treatment and clinical outcomes (1–5). High-throughput multi-omics and pharmacological profiling of cancer cell lines have been instrumental in characterizing molecular features which may aid drug–response predictions (4–6). Pioneer large screening cancer drug response datasets such as CCLE (7) and GDSC (8) have enabled scientists to gain more insight into pharmacogenomics via computational techniques for biomarker discovery and machine learning models for drug response predictions.

In recent years, several biomarkers were computationally identified based on the information from the large-scale cancer drug screening on hundreds of cancer cell lines (11–13). Due to the complexity of drug response mechanisms (14,15), various machine learning techniques have been used for predicting drug response based on omics profiles (16–20). Increasingly complex analysis techniques such as multimodal learning (21–23), visible machine learning (24), domain adaptation (25–27), and graph representation (28–30) have been explored. While most advancements in biomarker identification and machine learning models have focused on omics profiles, cancer drug sensitivity scores precalculated by the original sources are often used as-is, without consideration for variabilities between studies.

Commonly used scores include IC_50_ (a drug concentration that inhibits 50% of the cancer cells) or AUC (an area under the dose-response curve) values, which are inferred from multiple dose-response data points. Due to differences in experimental setups and preprocessing methods used to obtain the sensitivity scores, it is well-known that significant inconsistencies exist between the sensitivity scores across datasets (31–34). As a result, many studies opt to focus only on a single dataset, leading to the under-utilization of available data and a limited interpretation of cancer pharmacogenomics analysis.

Existing studies have attempted to mitigate the inconsistency using different strategies such as discretizing drug sensitivity levels (35,36), considering shared drug concentration ranges across multiple datasets (37), or investigating genetic and transcriptional evolutions that alter drug response in cancer cell lines (38). A few existing tools, such as PharmacoDB (39) and CellMinerCBD (40), have integrated multiple datasets and standardized the preprocessing methods for dose-response curves. However, the dose-response curve was processed separately for each dataset. Additionally, because an integrative summary of the dose-response curve across datasets was not provided, the variations (or uncertainties) of sensitivity scores at a specific drug dosage could not be captured.

To overcome these caveats, we have developed CREAMMIST, an integrative database that enables users to obtain an integrative dose-response, capturing uncertainty (or high certainty when multiple datasets are well aligned) across five widely used cancer cell-line drug–response datasets. Instead of estimating a dose-response curve for each dataset separately, we utilized the Bayesian framework to systematically integrate all available dose-response data points across datasets. The integration at the dose-response level provides an extended view of the dose-response curve to users, especially when different ranges of drug dosages were used across experiments.

We envision that CREAMMIST would suit various user types. Users can utilize easy-to-use statistics derived from the integrative dose-response curves for various downstream analyses such as identifying biomarkers, selecting drug concentrations for their experiments, and training robust machine learning models. An ability to visualize and extract the variations of the drug response across multiple datasets allows users to anticipate variability at various dosages and formulate their experiments accordingly.

## MATERIALS AND METHODS

### Data collection and processing

**Figure 1.**
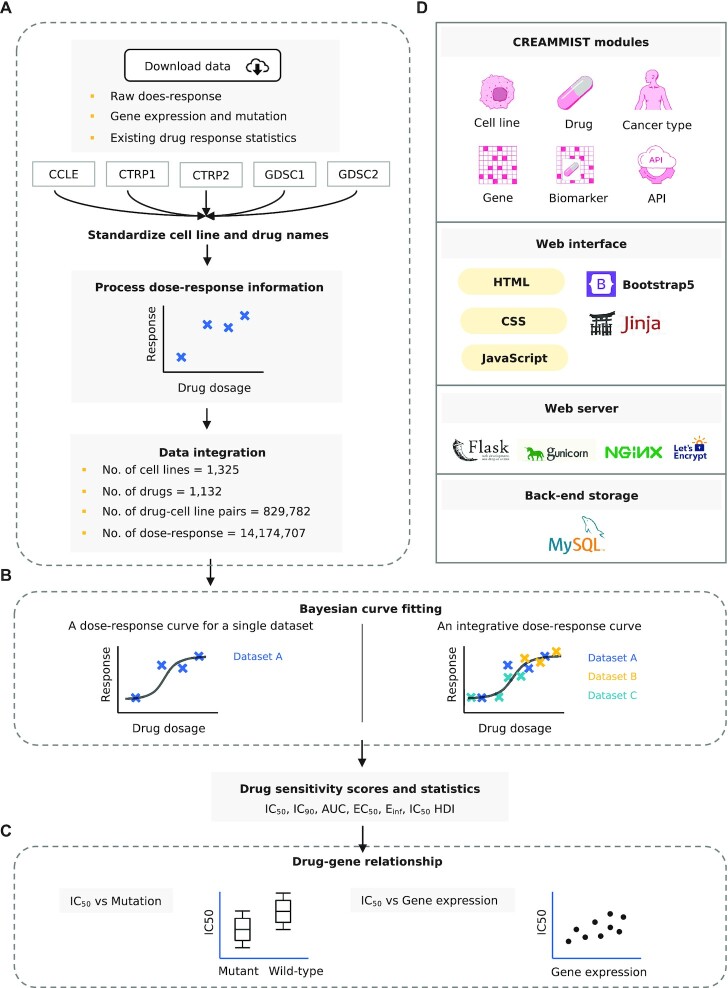
Flow chart of CREAMMIST. (**A**) Data collection and processing. Raw dose-response information, gene expression, and mutation profile are downloaded from CCLE, GDSC, and CTRP datasets. Cell line and drug names are standardized. Raw dose-response values are normalized and integrated into the database. (**B**) Bayesian curve fitting for an integrative dose-response curve. Multiple drug response statistics are calculated for each drug-cell line pair. (**C**) Quantifying the effect size of mutation on drug response (IC_50_ values) and calculating the correlation between gene expression level and drug response (IC_50_ values). (**D**) Storage and infrastructure of CREAMMIST.

Raw dose-response and omic profiles were downloaded and standardized for data downstream integrative analysis (Figure 1A). Raw dose-response data and precalculated IC_50_ and AUC values were downloaded from CCLE (9), GDSC (10) and CTRP (41). Gene expression and mutation data were downloaded from Cell Model Passport (42). For gene expression, we used TPM normalized values. For mutation, non-silent mutations in coding regions with allele frequency of at least 0.2 were selected for analysis. All cell line names were standardized based on Cellosaurus database (43), and drug names were manually curated by considering drug synonyms.

For each dataset, drug concentration is in $\mu$M unit, and response values at different concentrations were standardized in the range of 0 to 1 with respect to positive and negative control wells (Supplementary Figure S1), where 0 indicates no response (i.e. the same response level as negative control) and 1 indicates complete inhibition (i.e. the same response level as positive control). In total, CREAMMIST contains 1325 cell lines from 31 cancer types, 1132 drugs, 829 782 pairs of cell lines and drugs, and 14 174 707 dose-response data points (Supplementary Figure S2).

### Database organization

We used a relational database as a backend data storage to maintain both drug response and genotype information, as well as cell lines, drugs, and genes information. The database schema was designed to support multiple query types and identify candidate biomarkers using the original drug response score or the newly calculated drug response score based on the integrative dose-response curves (Supplementary Figure S3).

### Integration of dose–response data

To obtain an integrative dose–response curve across datasets for each pair of drug and cell line, we applied Bayesian curve fitting by using PyJags v1.3.7 (44) for the inference (Figure 1B). A commonly used logistic equation is defined as $f( x ) = \frac{1}{{1 + {2}^{ - k( {x - a} )}}}$, where $x$ represents drug concentration, $a$ represents the center position of the dose-response curve, and $k$ is the slope of the dose–response curve.

For the inference, we conducted three chains, where we performed 5000 iterations for each chain with 500 burn-in iterations. Different types of distributions were used for each parameter as follows. For robust estimation of the response value $f( x )$, we used a student's t-distribution $dt( {\frac{1}{{1 + {2}^{ - k( {x - a} )}}},\ {{( {\frac{1}{\sigma }} )}}^2,\ \nu } )$ with $\nu \sim dnorm( {250,\ 0.001} )$ and $\sigma \sim dunif( {0.01,\ 0.1} )$ as priors. The center position $a$ that represents IC_50_ was estimated using a normal distribution $a \sim dnorm( {{a}_\mu ,\ {{( {\frac{1}{{{a}_\sigma }}} )}}^2} )$ with ${a}_\mu \sim dnorm( {0,\ 1} )$ and ${a}_\sigma \sim dunif( {0.01, 5} )$ as priors. A non-negative slope value was estimated using a gamma distribution, $k \sim dgamma( {{k}_r,\ {k}_\lambda } )$ with ${k}_\lambda \sim dnorm( {2,\ 0.01} )$ and ${k}_r \sim dnorm( {1,\ 0.01} )$ as priors.

Multiple statistics, including mode and high-density interval (95% HDI) of parameters $a$ and $k$, as well as mean absolute error of the fitting, were obtained from the Bayesian fitted curves using Arviz v0.11.4, a Python package for exploratory analysis of Bayesian models (45). In addition to IC_50_, which corresponds to the center position of the curve, CREAMMIST also provides a drug concentration required for 90% inhibition of cell growth (IC_90_), area under the dose-response curve (AUC), a drug concentration at which 50% of its maximal response is induced (EC_50_), and maximum response caused by a drug (E_inf_).

### Identifying biomarkers from integrative dose-response information

To provide an overview of relationships between drug and gene, we compared IC_50_ obtained from the integrative curve against mutation and gene expression information of all (or cancer type specific) cell lines tested for each drug (Figure 1C). The mutational effect of each gene on drug response was calculated by using the Wilcoxon rank-sum test to compare IC_50_ values between wild-type and mutant cell lines. Wilcoxon rank-sum test was only performed for drug–gene pairs with at least ten wild-type and 10 mutant cell lines. The effect of gene expression level on drug response was calculated using Spearman correlation to avoid the extreme correlation value for some drug–gene pairs with outliers. Similar to the mutation data, the correlation was only calculated for drug–gene pairs with at least ten cell lines. To avoid the correlation calculation for noisy background gene expression values, we also required that at least five cell lines have at least one transcript per million (TPM).

### Implementation and web interface

All standardized dose-response data, integrative dose-response curves, omics profiles, and biomarkers information were stored in MySQL v8.0.30 (Figure 1D). CREAMMIST web application was deployed using Gunicorn v20.1.0, a Python Web Server Gateway Interface HTTP server. We used Nginx v1.18.0 as a proxy server and secured the connection with Let's Encrypt via certbot v0.40.0.

We used Flask v2.0.3 as a web application framework to implement CREAMMIST modules, including Cell lines, Drugs, Cancer types, Genes, and Biomarkers. We also utilized extensions such as Flask-Migrate v3.1.0 for database migration, Jinja2 v3.0.3 for HTML template management, and jQuery v1.12.1 for keyword suggestion. To enable a fully responsive web interface for both mobile and desktop users, we used Bootstrap v5.1.3. In addition to accessing the database via the web interface, we provide an Application Programming Interface (API), which allows other applications to access CREAMMIST.

## DATABASE CONTENT AND USAGE

### Integrative dose-response curve

**Figure 2.**
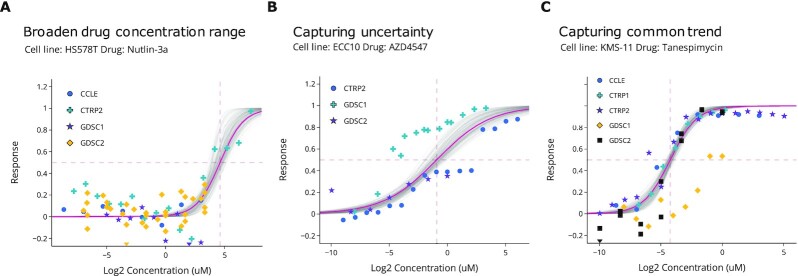
Integrative dose-response curves, where x-axis represents drug concentration and y-axis represents drug response level compared to the respective controls. Each color represents a dataset. Grey lines represent multiple curves resulting from Bayesian curve fitting, and a pink line represents the integrative dose–response curve based on mode values, i.e. location and slope parameters of a logistic function. Integrative dose–response curves capture (**A**) broaden concentration range by combining all dose-response data points across multiple datasets (**B**) uncertainty at different concentration levels (**C**) common trends across multiple datasets in the presence of discordance between datasets.

The key component of CREAMMIST is the integrative dose–response curve using Bayesian curve fitting. Fitting the dose–response curve independently for each dataset might lead to inconsistency of IC_50_ values. For instance, when the predetermined drug concentration range is on the lower end, we might not observe the inhibition effect and, consequently, IC_50_ is either not defined or too high because of the extrapolation. In contrast, the integrative dose–response curve consists of standardized dose–response data points from all available datasets for a given drug-cell line pair (Figure 2). Fitting a curve based on all dose–response data points allows users to view the response at a broader drug concentration range (Figure 2A). Moreover, using probabilistic curve fitting allows users to inspect the uncertainty at different drug concentrations (Figure 2B, grey lines) and capture the common trend in the presence of discordance between datasets (Figure 2C).

Although CREAMMIST allows comprehensive views of drug response across multiple datasets, there are some limitations of the integrative dose-response curves obtained from Bayesian curve fitting. As the fitted dose-response curves start at 0 (no response) and rise to 1 (complete inhibition), for highly resistant cases, drug sensitivity scores such as IC_50_ can be relatively high because the scores rely on extrapolation. In addition, the inferred curves might not well capture actual response values for a few outlier cases. Therefore, depending on the studies’ aims, users may consider high-density intervals of the sensitivity scores or mean absolute errors of the fitted curves to select drug-cell line pairs for their analysis.

### User interface modules

**Figure 3.**
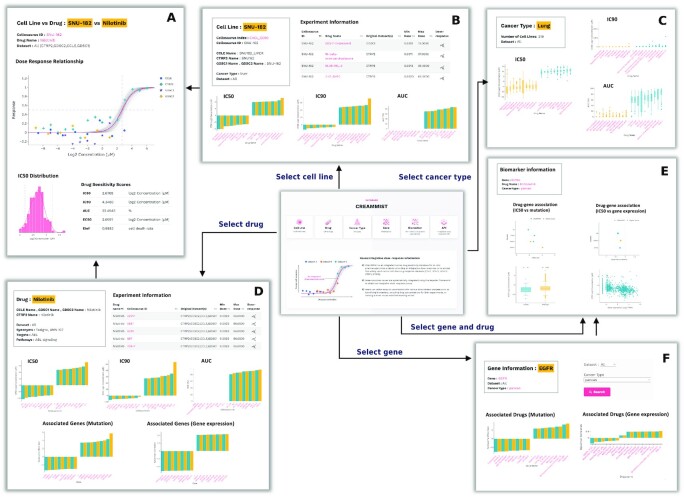
Overview of CREAMMIST web interface. (**A**) Interactive visualization of the integrative dose-response curve for a given drug-cell line pair. Based on the Bayesian curve fitting results, CREAMMIST provides IC_50_ distribution and multiple drug sensitivity scores. (**B**) Cell line module provides cell line information and relevant drug screening experiment information. (**C**) Cancer type module provides overall information across all cell lines belonging to a given cancer type. (**D**) Drug module provides drug information and relevant drug screening experiment information. (**E**) Biomarker module contains drug–gene associations based on mutation and gene expression. (**F**) Gene module provides gene information and relevant drug–gene associations.

The front page of CREAMMIST explains the concept of the integrative dose-response curve and allows users to browse through the database from different perspectives (Figure 3). CREAMMIST provides interactive plots for users to view the integrative dose-response curve, IC_50_ distribution inferred from the Bayesian curve fitting, and five drug sensitivity scores based on the integrative dose-response curve (Figure 3A). Users can select the following modules to start searching or browsing. Information on every webpage is downloadable in a text format, and plots are downloadable in SVG format.

### Cell line module

Users search or browse the database by cell line name. The cell line page shows cell line information alongside relevant drug screening experiments and top drugs based on various sensitivity scores (Figure 3B). For each experiment, users can extract more detail and visualize the integrative dose-response curve.

### Cancer type module

To study cancer-specific drug response, users can select the cancer type of interest to retrieve drug response information. Users can also select to view either the sensitivity scores based on the integrative dose-response curve or the provided scores from the original data sources (Figure 3C).

### Drug module

Users search or browse the database by drug name. The drug page shows drug information alongside relevant drug screening experiments and top cell lines based on various sensitivity scores (Figure 3D). Similar to the cell line module, users can extract more detail and visualize the integrative dose-response curve.

### Biomarker module

CREMMIST precalculated the effect of genetic alteration, including point mutations and changes in gene expression level, on each drug. Users can search a gene-drug pair to view the details, and the interactive plots allow users to inspect the values of each data point (Figure 3E). Users can also select cancer types to extract the effects calculated based only on cell lines from a given cancer type.

### Gene module

Users search or browse the database by gene name. The gene page shows gene information alongside relevant drugs based on the effect scores (Figure 3F). For each drug, users can extract more detail and inspect the gene–drug relationships through the Biomarker module.

## SUMMARY AND FUTURE DIRECTIONS

We systematically integrated dose-response data from multiple data sources, allowing users to retrieve the integrative dose-response curves. CREAMMIST provides easy-to-use statistics, interactive plots, and API for programmatically accessing the database consisting of more than 14 millions dose-response data points. Instead of attempting to remove the unavoidable inconsistency between studies or even among replicates, we applied a Bayesian framework for dose-response curve fitting to capture the uncertainties at different drug concentration levels. Users can obtain drug response information from broader drug concentration ranges and, at the same time, be able to anticipate variability at various dosages and formulate their experiments accordingly.

CREAMMIST can be useful for various downstream analyses such as identifying biomarkers, selecting drug concentrations for future experiments, and training a more robust machine learning model by incorporating confidence interval information. Going forwards, CREMMIST can be enhanced by: (a) incorporating chemical properties of the drugs, enabling users to search for similar drugs based on both response profiles and chemical similarity; (b) applying matrix factorization, a technique used in recommender systems, to facilitate users to discover similar drugs (or cell lines or genes); (c) taking into account additional experimental setup information such as durations and different types of controls for more advance search and analysis; (d) integrating other types of omics profiles; and (e) allowing users to incorporate their in-house experimental results such as drug screening on patient-derived cell lines.

Despite advances in the pharmacogenomic field and sequencing technologies, less than a quarter of oncology patients benefit directly from precision medicine (46,47). Most treatments still rely on invasive, toxic standard therapies because physicians had no way to reliably predict which drug would work best for each patient (48). Diverse strategies to make use of the existing cancer drug screening data are needed not only for biomarker identification but also for building knowledge to guide drug repropositioning (11,49). As inter- and intra-tumor heterogeneity pose further challenges to cancer treatments, the diversity of cancer cell lines with drug response information could also reveal distinct drug response behaviors across subpopulations (50,51) as well as novel target populations (52).

## Supplementary Material

gkac911_Supplemental_File

## Notes

Present address: Chayaporn Suphavilai, Genome Institute of Singapore, A*STAR Research Institute, 60 Biopolis Way, 138672 Singapore.
