# Supplementary material for: CREAMMIST: an integrative probabilistic database for cancer drug response prediction
Source: Nucleic Acids Res. 2022 Oct 19;51(D1):D1242–8. doi: 10.1093/nar/gkac911 (PMC9825458; doi:10.1093/nar/gkac911)
Supplement: gkac911_Supplemental_File [file gkac911_supplemental_file.pdf]

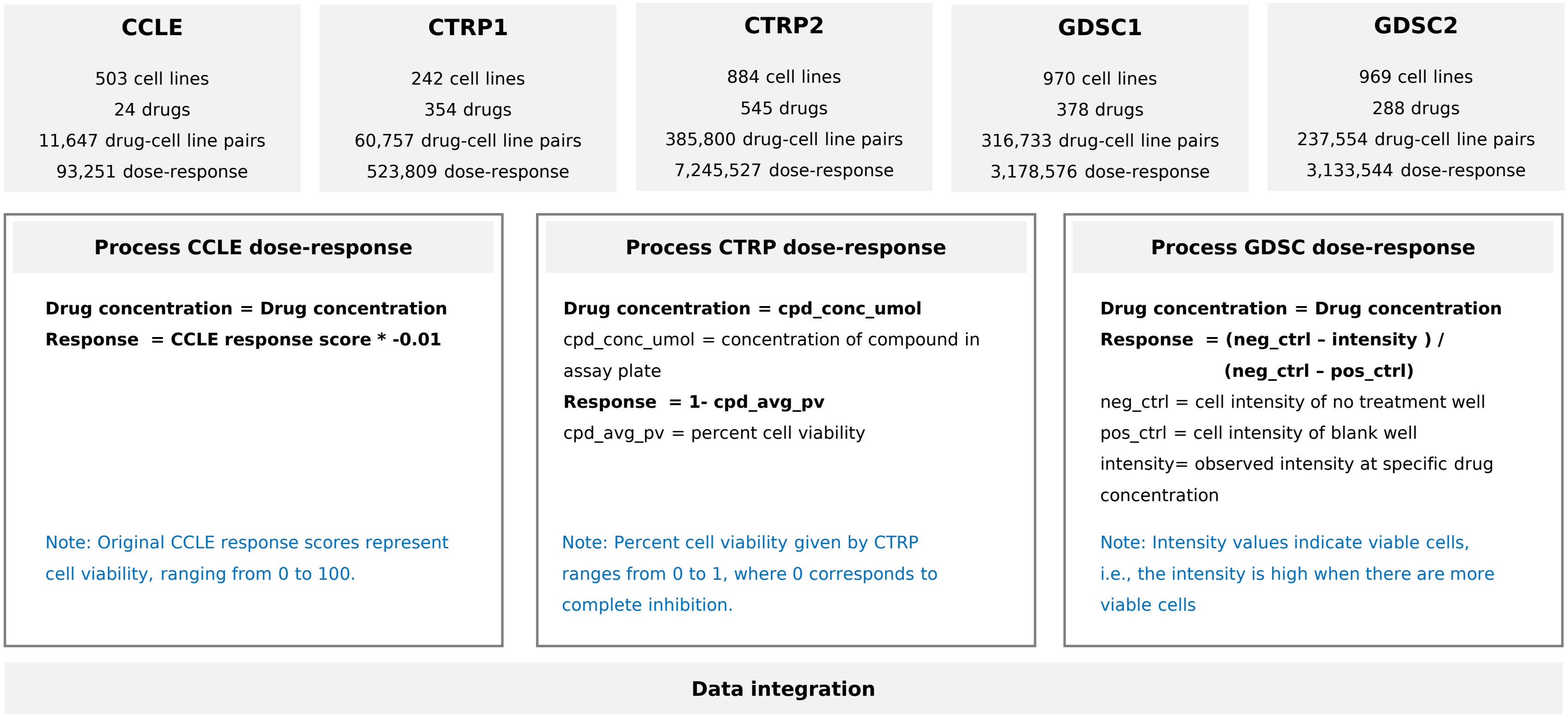

Supplementary Figure 1. Standardization of drug response values for each dataset. All raw response scores from different datasets were standardized into the range of 0 to 1, where 0 represents 0% cell inhibition (cell death) compared to the respective controls and 1 represents complete inhibition.

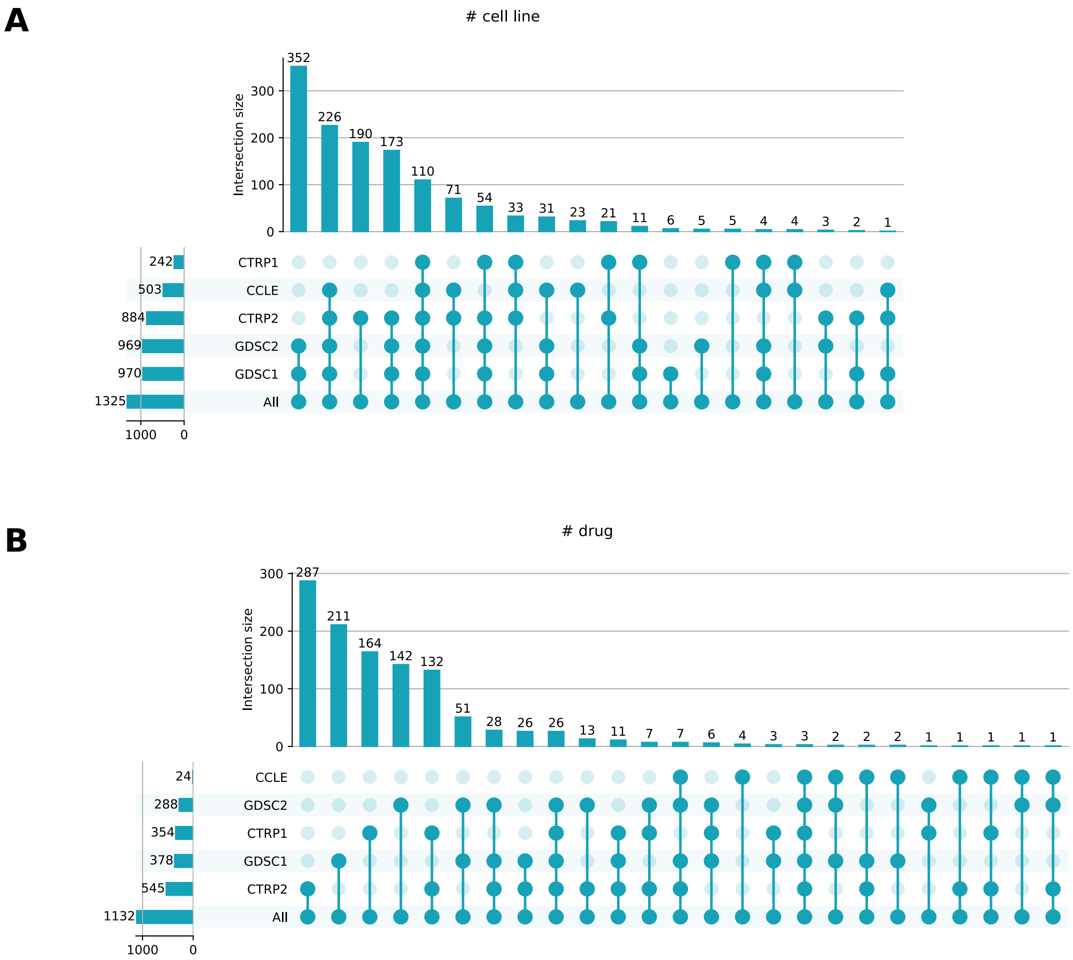

Supplementary Figure 2. Overall statistics. (A) Number of unique and common cell lines across five datasets. (B) Number of unique and common drugs across five datasets.

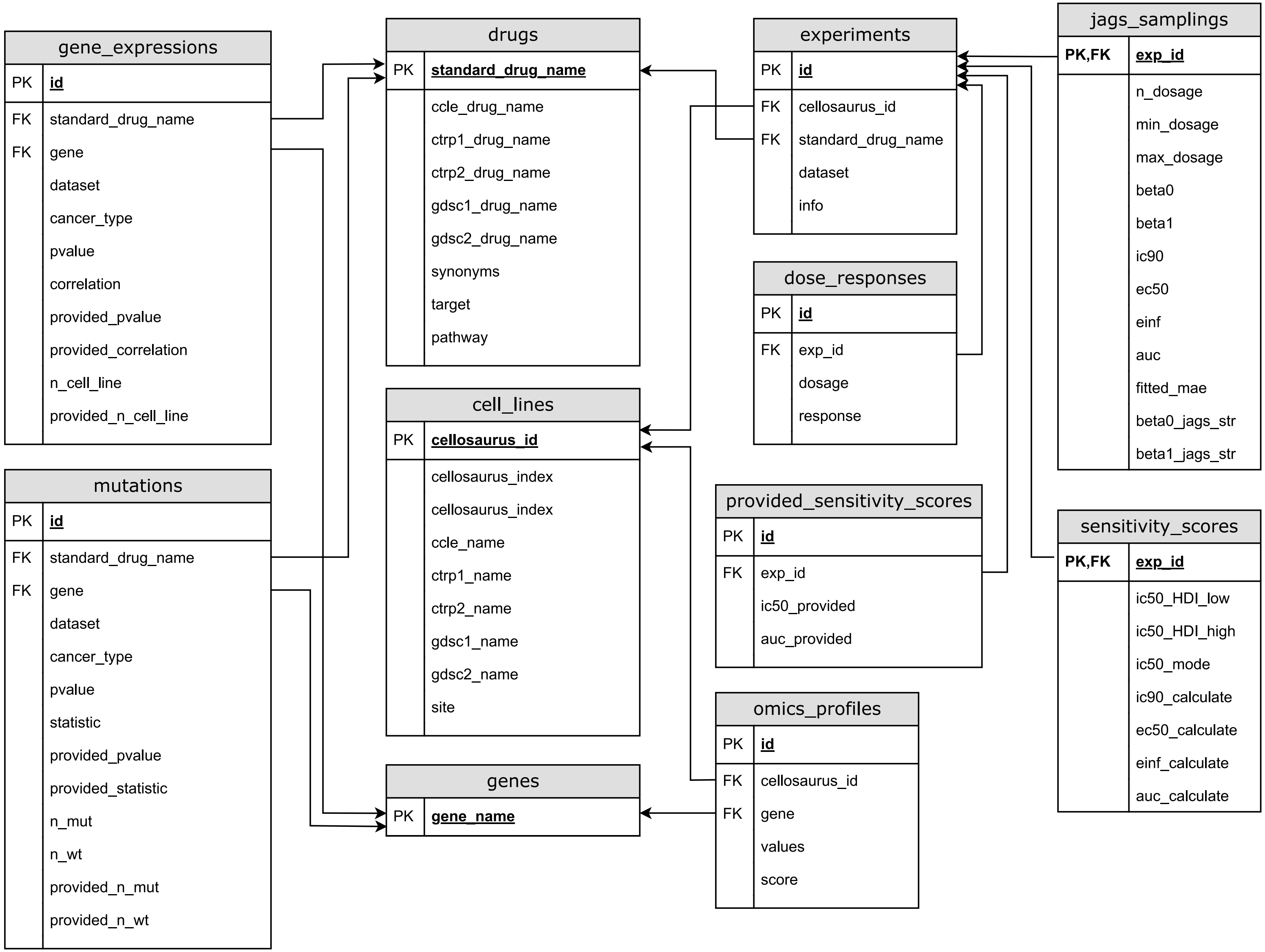

Supplementary Figure 3. CREAMMIST database schema.
